# Reaction Mechanism Study of LiNH_2_BH_3_ and (LiH)_n_ (n = 1–5) Clusters Based on Density Functional Theory

**DOI:** 10.3390/molecules30040929

**Published:** 2025-02-17

**Authors:** Xiao Dong, Rong Yuan, Genzhuang Li, Aochen Du

**Affiliations:** Xinjiang Laboratory of Phase Transitions and Microstructures in Condensed Matters, College of Physical Science and Technology, Yili Normal University, Yining 835000, China; dx357@126.com (X.D.); 17793214291@163.com (R.Y.); 24214@ylnu.edu.cn (G.L.)

**Keywords:** hydrogen storage materials, density functional theory, transition states

## Abstract

Hydrogen energy is an ideal clean energy source for the future. In the promotion and application of hydrogen energy, the safe and effective storage of hydrogen needs to be addressed. LiNH_2_BH_3_, as an important hydrogen storage material, can reversibly store hydrogen, but it has the problem of a relatively high hydrogen release temperature. (LiH)_n_ plays a good regulatory role in the metal–N–H system and plays an important role. Using density functional theory, the reaction mechanism of LiNH_2_BH_3_ and (LiH)_n_ (n = 1–5) clusters was theoretically calculated and analyzed. The frontier orbitals of LiNH_2_BH_3_ (LiAB), LiNH_2_BH_3_–LiH (Li2AB), and LiNH_2_–LiH (Li2A) were compared and analyzed, and the dissociation energies of hydrogen atoms at different sites were discussed. The results show that the dehydrogenation of LiNH_2_BH_3_ with (LiH)_n_ (n = 1–5) clusters is more likely to occur through the combination of H^δ−^(Li)···H^δ+^(N), and the minimum reaction energy barrier can reach 113.34 kJ/mol. In the LiNH_2_BH_3_–LiH system, the presence of –BH_3_ and –LiH groups has a significant effect on the hydrogen release performance of the system. The order of hydrogen atom dissociation energies at different positions in LiAB, Li2AB, and Li2A is ΔE_H(N)_ > ΔE_H(B)_ > ΔE_H(Li)_. The dehydrogenation performance of Li2AB is better than that of LiAB and Li2A.

## 1. Introduction

Energy assumes a vital role in the development of human society and pertains to all facets of people’s lives. At present, as society is undergoing rapid development, it is concurrently accompanied by the continuous aggravation of energy problems and issues like climate change, which have presented significant impediments to the sustainable and green development of humanity [1,2]. Hydrogen energy is regarded as a highly promising clean energy source. Achieving high-weight and reversible hydrogen storage is a problem that needs to be addressed [3].

Hydrogen storage materials play a crucial role in the advancement of hydrogen energy technologies, which are increasingly recognized as a promising solution to the global energy crisis and environmental challenges [4]. As a clean and renewable energy source, hydrogen has the potential to significantly reduce greenhouse gas emissions and reliance on fossil fuels. However, the widespread adoption of hydrogen as an energy carrier is hindered by the challenges associated with its safe and efficient storage [5]. Solid–state chemical hydrogen storage using hydrogen storage materials as the medium has a promising application prospect due to its high safety and hydrogen storage density. The solid–state chemical hydrogen storage method utilizes hydrogen storage materials as a medium, enabling the safe reversible storage and release of hydrogen, and has a high hydrogen storage density [6]. Solid–state chemical hydrogen storage materials mainly include organic porous hydrogen storage materials [7,8], carbon-based hydrogen storage materials [9,10], metal-based hydrogen storage materials [11,12,13], and coordination hydride hydrogen storage materials [14,15,16,17]. Among them, the first two belong to physical adsorption hydrogen storage methods, mainly having the problem of low hydrogen storage capacity; the latter two belong to chemical hydrogen storage methods, mainly having the problem of high hydrogen release temperature in terms of hydrogen storage and release kinetics. Current research on hydrogen storage materials primarily emphasizes optimizing hydrogen storage capacity and release temperatures. Among various hydrogen storage materials, NH_3_BH_3_ has emerged as a leading candidate due to its high hydrogen content, theoretically capable of releasing up to 19.6 wt% of hydrogen [18]. However, the practical application of NH_3_BH_3_ is hindered by its high dehydrogenation temperature, typically exceeding 100 °C, and the concomitant production of impurity gases such as B_2_H_6_ and NH_3_ [19]. This temperature is higher than the working temperature of about 90 °C of a polymer electrolyte membrane (PEM) fuel cell. At higher temperatures, the evaporation of water will seriously reduce the conductivity of a PEM. To actually use the NH_3_BH_3_ complex as a hydrogen source for PEM fuel cells, it is necessary to lower the dehydrogenation temperature of this complex [20].

To address these limitations, researchers have turned to metal borohydrides, particularly metal ammonia boranes, such as LiNH_2_BH_3_ [21,22]. LiNH_2_BH_3_ has attracted extensive attention due to its outstanding hydrogen storage performance. It can release 10.9 wt% of hydrogen at a temperature below 100 °C without generating harmful by–products like borazine, and is regarded as one of the most promising materials for on–board hydrogen storage applications [23]. The incorporation of metal ions into the ammonia borane framework has been shown to significantly reduce the dehydrogenation temperature, thus improving the kinetics of hydrogen release [24]. Furthermore, the presence of metal ions can stabilize the ammonia borane structure, effectively suppressing the generation of impurity gases during the dehydrogenation process [25]. LiH has also been identified as a crucial component in the metal–N–H systems [26,27]. Lee et al. [28] performed a theoretical investigation on the reaction between (LiH)_4_ and NH_3_BH_3_ leading to the formation of LiNH_2_BH_3_, followed by systematic examination of the dehydrogenation processes. Their study specifically addressed two distinct mechanisms: (1) the dehydrogenation pathway of the resulting (LiH)_3_·LiNH_2_BH_3_ complex, and (2) the sequential hydrogen release mechanism through (LiNH_2_BH_3_)_2_ dimer formation that ultimately generates four H_2_ molecules. The research revealed two competing dehydrogenation mechanisms: a concerted N–B bond-mediated pathway and an alternative pathway involving hydride transfer from boron to nitrogen via Li+ mediation, which facilitates the formation of an Li–H–Li bridge intermediate. Notably, the latter mechanism exhibited a significantly lower energy barrier compared to the concerted pathway. This comparative analysis demonstrated the crucial catalytic role of Li^+^ ions in enabling hydride ion transport between heteroatoms, thereby significantly enhancing the hydrogen release kinetics. LiH possesses unique properties that allow it to play a pivotal role in regulating the hydrogen release behavior of ammonia boranes. Its ability to form stable complexes with ammonia boranes can further lower the dehydrogenation temperature and improve the overall hydrogen release performance [28]. Given that LiH incorporation significantly enhances the dehydrogenation performance of NH_3_BH_3_, coupled with the lower energy barrier observed in the Li^+^-mediated hydride transfer mechanism through Li–H–Li bridge formation in (LiNH_2_BH_3_)_2_ dimers, this study strategically introduces (LiH)_n_ clusters (n = 1–5) into the LiNH_2_BH_3_ system. This configuration enables dual pathways for H_2_ generation through acid–base paired H^δ−^···H^δ+^ interactions: (1) H^δ−^(B)···H^δ+^(N) coupling and (2) H^δ−^(Li)···H^δ+^(N) association. Notably, the latter pathway directly utilizes the intrinsic Li–H bonds present in native LiH clusters, bypassing the conventional requirement for Li^+^-mediated hydride transfer from boron centers to form transient Li–H–Li bridges. A comparative analysis was conducted across three distinct systems: pristine LiNH_2_BH_3_, LiNH_2_BH_3_–LiH composites, and LiNH_2_–LiH configurations. This systematic comparison reveals that the coexistence of –BH_3_ and –LiH functional groups in the LiNH_2_BH_3_–LiH hybrid system synergistically optimizes hydrogen release characteristics. The –BH_3_ groups primarily facilitate proton donor capabilities through N–H^δ+^ sites, while the –LiH components provide enhanced hydride mobility via Li–H^δ−^ interactions, collectively establishing an efficient dual–channel dehydrogenation framework.

In conclusion, considering that the addition of LiH can significantly improve the hydrogen release performance of NH_3_BH_3_ and also plays an important role in the metal–N–H system, LiH is further introduced on the basis of LiNH_2_BH_3_ to discuss its promoting effect on the dehydrogenation of LiNH_2_BH_3_. The research on the reaction mechanism of LiNH_2_BH_3_ and (LiH)_n_ (n = 1–5) clusters is intended to improve the hydrogen release performance and simultaneously minimize the production of impurity gases. The insights obtained from computational studies will not only deepen comprehension of the underlying mechanisms but also offer inspirations for the research directions of future efficient hydrogen storage solutions. As the world progresses towards a more sustainable energy future, the development of innovative hydrogen storage materials is of crucial significance for fully exploiting the potential of hydrogen as a clean energy carrier.

## 2. Results

### 2.1. Reaction Between LiNH_2_BH_3_ and LiH Clusters

**Figure 1:**
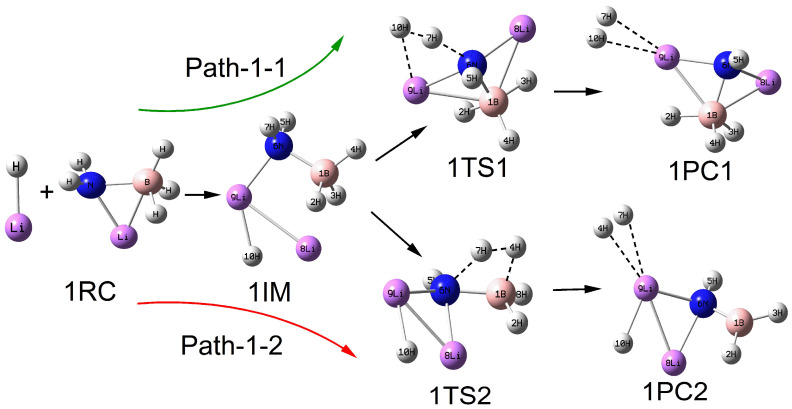
Geometries of the critical points of the potential energy surface of the reaction between LiNH_2_BH_3_ and LiH.

As depicted in Figure 1, the configurations of the relevant stationary points on the potential energy surface of the reaction between LiNH_2_BH_3_ and LiH are presented. The reactants LiNH_2_BH_3_ and LiH come together to form the intermediate 1IM. The natural charge distribution of 1IM was determined using the natural bond orbital (NBO) method. It was found that the H atom connected to the B atom shows a negative charge, denoted as H^δ−^(B), with a charge of −0.028 to −0.096; the H atom connected to the N atom shows a positive charge, denoted as H^δ+^(N), with a charge of 0.403; and the H atom connected to the Li atom shows a negative charge, denoted as H^δ−^(Li), with a charge of −0.593. Based on this, the dehydrogenation reaction path of LiNH_2_BH_3_ and LiH is designed into two reaction paths: H^δ−^(Li)···H^δ+^(N) combined dehydrogenation and H^δ−^(B)···H^δ+^(N) combined dehydrogenation. Meanwhile, since the charge of H^δ−^(Li) is significantly greater than that of H^δ−^(B), it can be judged that the potential interaction between H^δ−^(Li)···H^δ+^(N) is greater than that between H^δ−^(B)···H^δ+^(N), indicating that the reaction path of H^δ−^(Li)···H^δ+^(N) combined dehydrogenation is easier to proceed.

The dehydrogenation reaction path of H^δ−^(Li)···H^δ+^(N) combined is represented as follows: 1RC→1IM→1TS1→1PC1, denoted as Path–1–1. The process from the intermediate 1IM to the transition state 1TS1 mainly corresponds to the gradual separation of H(7) from the N(6) atom, with the bond length increasing from 0.1019 nm to 0.1382 nm; the distance between H(7) and H(10) continuously decreases to 0.1001 nm. In the subsequent process of forming the product 1PC1 from the transition state 1TS1, the distance between H(7) and H(10) further decreases to 0.0747 nm, which reflects the characteristic of a hydrogen molecule, and finally the hydrogen molecule stably adsorbs on the top position of the Li atom. The dehydrogenation reaction path of H^δ−^(B)···H^δ+^(N) combined is expressed as follows: 1RC→1IM→1TS2→1PC2, denoted as Path–1–2. The process from the intermediate 1IM to the transition state 1TS2 mainly corresponds to the increase in the distance between H(7) and N(6) atoms, with the bond length increasing from 0.1019 nm to 0.1500 nm; the increase in the distance between H(4) and B(1) atoms, with the bond length increasing from 0.1210 nm to 0.1394 nm; and the continuous decrease in the distance between H(7) and H(4) to 0.0959 nm. Subsequently, in the process of forming the product 1PC2 from the transition state 1TS2, the distance between H(7) and H(4) further decreases to 0.0747 nm, and finally the hydrogen molecule also stably adsorbs on the top position of the Li atom.

### 2.2. Reaction Between LiNH_2_BH_3_ and (LiH)_n_ (n = 2–5) Clusters

**Figure 2:**
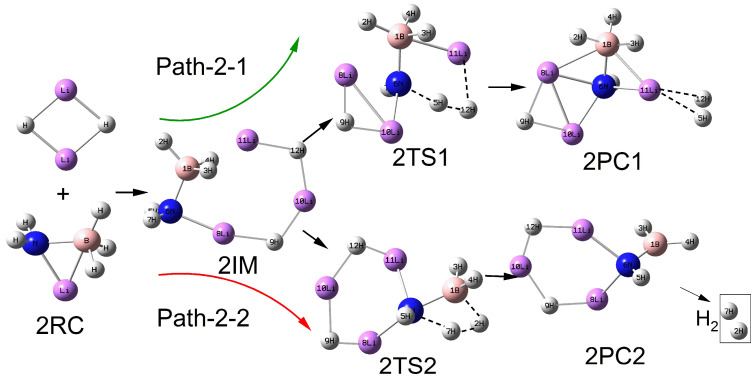
Geometries of the critical points of the potential energy surface of the reaction between LiNH_2_BH_3_ and (LiH)_2_.

**Figure 3:**
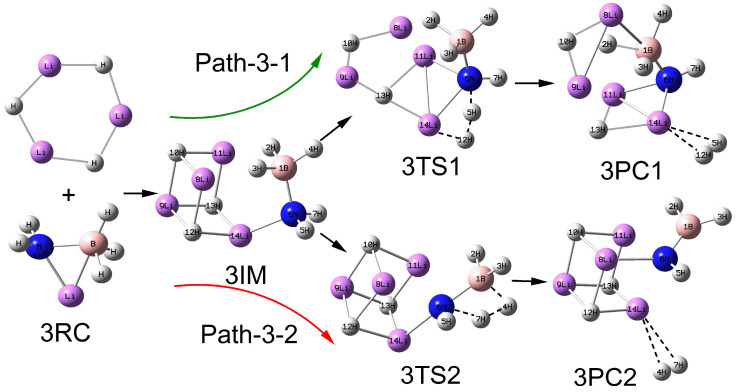
Geometries of the critical points of the potential energy surface of the reaction between LiNH_2_BH_3_ and (LiH)_3_.

**Figure 4:**
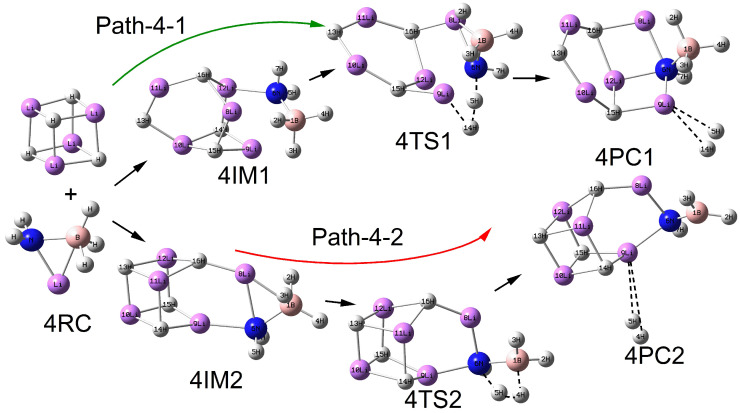
Geometries of the critical points of the potential energy surface of the reaction between LiNH_2_BH_3_ and (LiH)_4_.

**Figure 5:**
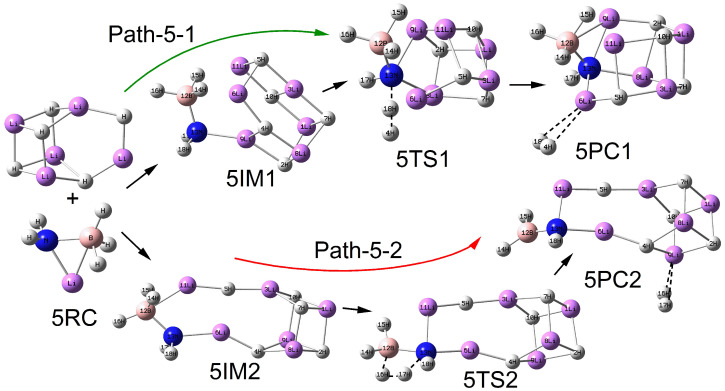
Geometries of the critical points of the potential energy surface of the reaction between LiNH_2_BH_3_ and (LiH)_5_.

As shown in Figure 2, Figure 3, Figure 4 and Figure 5, the configurations of the relevant stationary points on the potential energy surface of the reaction between LiNH_2_BH_3_ and (LiH)_n_ (n = 2–5) are presented. Similar to the reaction process of LiH, the reaction processes of LiNH_2_BH_3_ and (LiH)_n_ (n = 2–5) both correspond to two reaction pathways: dehydrogenation through H^δ−^(Li)···H^δ+^(N) combination and dehydrogenation through H^δ−^(B)···H^δ+^(N) combination. As illustrated in Figure 1, Figure 2, Figure 3, Figure 4 and Figure 5, the dehydrogenation pathways involving the combination of H^δ−^(Li)···H^δ+^(N) are respectively denoted as Path–x–1 (x = 1–5), while those involving the combination of H^δ−^(B)···H^δ+^(N) are respectively denoted as Path–x–2 (x = 1–5).

From Figure 1, Figure 2, Figure 3, Figure 4 and Figure 5, it can be observed that after the dehydrogenation reaction of LiNH_2_BH_3_ and (LiH)_n_ (n = 1–5), the hydrogen molecules are almost all stably adsorbed at the top positions of Li atoms. Additionally, an interesting phenomenon can be noted: for the reactions of LiNH_2_BH_3_ and (LiH)_n_ (n = 1–5), the products resulting from dehydrogenation through H^δ−^(B)···H^δ+^(N) combination can be regarded as (LiH)_n+1_ clusters with one H atom replaced by the –NHBH_2_ functional group. For instance, the situation in product 3PC2 clearly reflects this. This indicates that after the dehydrogenation reaction of LiNH_2_BH_3_ with (LiH)_n_ (n = 1–5), Li tends to aggregate into LiH clusters. In addition, it is particularly noted that the Li–N and B–N bond lengths in the intermediate 3IM are 0.1964 nm and 0.1574 nm, respectively, which are in good agreement with the Li–N and B–N bond lengths of 0.1984 nm and 0.1571 nm in (LiH)_3_·NH_2_BH_3_ reported in the literature. Moreover, under the same dehydrogenation mechanism, the dehydrogenation energy barrier of (LiH)_3_·NH_2_BH_3_ is 51.4 kcal/mol (215.06 kJ/mol), which is highly consistent with the dehydrogenation energy barrier of 214.71 kJ/mol for the Path–3–2 reaction [28]. This indicates that the computational method and basis set adopted in this paper are applicable and the results are reliable. The clear configurations of the transition states on each reaction path are presented in the Supporting Information (Figure S1).

### 2.3. Analysis of Reaction Energy and Mechanism

**Figure 6:**
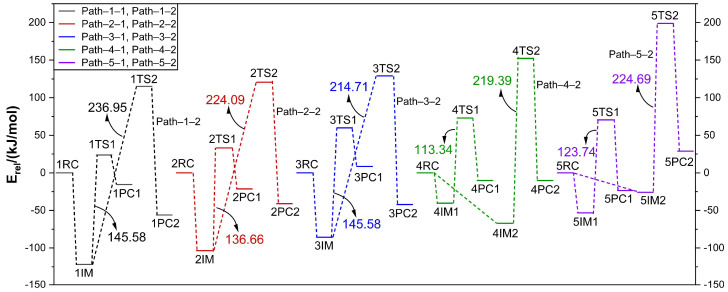
Energetic profiles for potential energy surface of each reaction.

From the above description of the dehydrogenation process of LiNH_2_BH_3_ and (LiH)_n_ (n = 1–5), it can be found that the dehydrogenation processes of each reaction have certain similarities. For the dehydrogenation process combined with H^δ−^(Li)···H^δ+^(N), the formation of the transition state corresponds to the increase in distance between the H and N atoms. While for the dehydrogenation process combined with H^δ−^(B)···H^δ+^(N), the formation of the transition state corresponds to the increase in distances between the H and N atoms and between the H and B. The increase and even the breaking of the N–H or B–H bond length requires overcoming a certain energy barrier, which reflects the difficulty of each reaction and is also the key to the dehydrogenation reaction. During the reaction process, there will be changes in energy, so the relationship between the energies of each stationary point in the reaction process plays an important role in describing the reaction mechanism. As shown in Table S1, the energy information of each reaction–related stationary point and some of the vibration frequencies are listed, where E_total_ is the total energy of the stationary point, and E_rel_ is the relative energy with respect to the total energy of each reactant. Each transition state has only one imaginary frequency, which indicates the correctness of the calculated transition state configuration. At the same time, an intrinsic reaction coordinate (IRC) analysis was further conducted for each transition state to determine the correctness of the connection relationship between the transition state and other stationary points. To more intuitively judge the energy changes in the reaction process, the heat absorption and release and the reaction energy barrier during the reaction process were analyzed; the potential energy surface profile diagram based on relative energy is given in Figure 6.

As shown in Figure 6, for the dehydrogenation reaction paths involving H^δ−^(Li)···H^δ+^(N), including Path–1–1, Path–2–1, Path–3–1, Path–4–1, and Path–5–1, the dehydrogenation energy barriers are 145.58, 136.66, 145.58, 113.34, and 123.74 kJ/mol, respectively. For the dehydrogenation reaction paths involving H^δ−^(B)···H^δ+^(N), including Path–1–2, Path–2–2, Path–3–2, Path–4–2, and Path–5–2, the dehydrogenation energy barriers are 236.95, 224.09, 214.71, 219.39, and 224.69 kJ/mol, respectively. The dehydrogenation energy barriers do not show a linear relationship with the size of the LiH cluster. However, it is obvious that the dehydrogenation reaction is more likely to occur through the H^δ−^(Li)···H^δ+^(N) combination, among which Path–4–1 has the lowest energy barrier, with a value of 113.34 kJ/mol. This result is consistent with the previous statement that due to the significantly larger charge of H^δ−^(Li) compared to H^δ−^(B), the potential interaction between H^δ−^(Li)···H^δ+^(N) is greater than that between H^δ−^(B)···H^δ+^(N), indicating that the dehydrogenation through H^δ−^(Li)···H^δ+^(N) is relatively easier. Another possible reason is that the dehydrogenation through H^δ−^(B)···H^δ+^(N) not only needs to overcome the breaking of the N–H bond but also the B–H bond, so the dehydrogenation energy barrier is relatively much higher.

**Figure 7:**
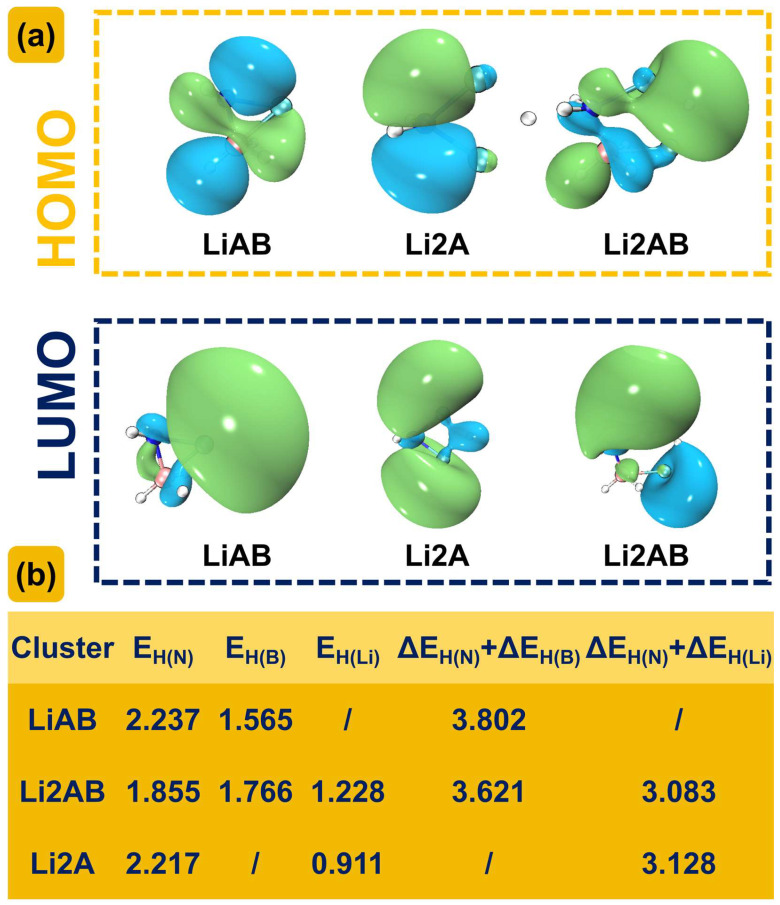
(**a**) Front–line orbital distributions of LiAB, Li2AB, and Li2A. (**b**) Hydrogen removal energies of LiAB, Li2AB, and Li2A (eV).

Our research group has previously conducted computational studies on the reaction mechanism of LiNH_2_ with LiH, and the energy barrier for dehydrogenation was found to be 239.8 kJ/mol. This value is significantly higher than that of the reaction between LiNH_2_BH_3_ and LiH (Path–1–1: 145.58 kJ/mol). This indicates that the presence of the –BH_3_ group has a considerable impact on the hydrogen storage material of the LiNH_2_–LiH system, reducing the energy barrier for dehydrogenation and improving the hydrogen storage and release performance. The energy barrier for dehydrogenation of LiNH_2_BH_3_ itself was calculated to be 262.81 kJ/mol. This process involves a combination of H^δ−^(B)···H^δ+^(N) dehydrogenation, but due to space limitations, it will not be elaborated in this paper. Compared with the reaction between LiNH_2_BH_3_ and LiH (Path–1–2: 236.95 kJ/mol), the energy barrier for dehydrogenation of LiNH_2_BH_3_ itself is higher, indicating that the introduction of LiH can improve the hydrogen release performance of LiNH_2_BH_3_. According to the frontier orbital theory proposed by Fukui Kenichi, the highest occupied molecular orbital (HOMO) and the lowest unoccupied molecular orbital (LUMO) of a molecule are crucial in determining the occurrence of chemical reactions. To understand the influence of the –BH_3_ group and the introduction of LiH on the LiNH_2_BH_3_–LiH system, the frontier orbitals of LiNH_2_BH_3_ (LiAB), LiNH_2_BH_3_–LiH (Li2AB), and LiNH_2_–LiH (Li2A) were calculated and analyzed. As shown in Figure 7a, the LUMO orbitals of LiAB, Li2AB, and Li2A are mainly distributed around the Li atoms, with the electron–accepting regions concentrated at the Li atoms. For the HOMO orbitals, compared with LiAB, the HOMO orbital of Li2AB shifts towards the –LiH region, while compared with Li2A, the HOMO orbital of Li2AB shifts towards both the –BH_3_ and –LiH regions. This indicates that the introduction of the –BH_3_ group and LiH has little effect on the LUMO orbitals but mainly affects the HOMO orbitals. The electron–donating regions of Li2AB are distributed at both the –BH_3_ and –LiH regions, which also corresponds to the two dehydrogenation pathways.

To more clearly illustrate that the dehydrogenation of LiNH_2_BH_3_ with LiH is more inclined to the dehydrogenation mode of H^δ−^(Li)···H^δ+^(N) combination, its dehydrogenation energy barrier (Path–1–1: 145.58 kJ/mol) is lower than that of the reaction between LiNH_2_ and LiH (239.8 kJ/mol), and the dehydrogenation energy barrier of LiNH_2_BH_3_ with LiH in the mode of H^δ−^(B)···H^δ+^(N) combination (Path–1–2: 236.95 kJ/mol) is lower than the self–dehydrogenation energy barrier of LiNH_2_BH_3_ (262.81 kJ/mol), the dissociation energies of hydrogen atoms at different positions of LiAB, Li2AB and Li2A were calculated, and the results are shown in Figure 7b. The dissociation energy of hydrogen atoms can be calculated via the following formula: ∆E_H(x)_ = E_0_(MH_n−1_) + 0.5E_0_(H2) − E_0_(MH_n_). ΔE_H(x)_ represents the dissociation energy of hydrogen atoms at different sites, E_0_[MH_n−1_] is the energy of the system after dissociating a hydrogen atom with zero–point energy correction, E_0_[H_2_] is the energy of H_2_ with zero–point energy correction, and E_0_[MH_n_] is the energy of the original system with zero–point energy correction. As shown in Figure 7b, the order of the dissociation energies of hydrogen atoms at different positions in LiAB, Li2AB, and Li2A is ΔE_H(N)_ > ΔE_H(B)_ > ΔE_H(Li)_, which also indicates the strength of N–H, B–H, and Li–H bonds. The ΔE_H(N)_ of Li2AB is smaller than that of Li2AB and Li2A, indicating that the N–H bond in Li2AB is more easily broken and more conducive to hydrogen release. The value of ΔE_H(N)_ + ΔE_H(B)_ of Li2AB (3.621 eV) is larger than the value of ΔE_H(N)_ + ΔE_H(Li)_ (3.083 eV), which first indicates that the dehydrogenation of LiNH_2_BH_3_ with LiH is more likely to occur through the H^δ−^(Li)···H^δ+^(N) combination dehydrogenation mode, consistent with the previous conclusion about the dehydrogenation energy barrier. Compared with LiAB, the ΔE_H(N)_ + ΔE_H(B)_ of Li2AB is smaller, indicating that the dehydrogenation energy barrier of LiNH_2_BH_3_ with LiH through the H^δ−^(B)···H^δ+^(N) combination is lower than that of LiNH_2_BH_3_ itself. Compared with Li2A, the ΔE_H(N)_ + ΔE_H(Li)_ of Li2AB is smaller, indicating that the dehydrogenation energy barrier of LiNH_2_BH_3_ with LiH through the H^δ−^(Li)···H^δ+^(N) combination is lower than that of LiNH_2_ with LiH.

## 3. Discussion

From the above discussion, it can be concluded that the dehydrogenation of LiNH_2_BH_3_ with (LiH)_n_ (n = 1–5) clusters is more inclined to the dehydrogenation mode of the H^δ−^(Li)···H^δ+^(N) combination, with the minimum reaction energy barrier reaching 113.34 kJ/mol. For the dehydrogenation reactions of LiNH_2_BH_3_–LiH (Path–1–1) and LiNH_2_–LiH, both follow the dehydrogenation mode of the H^δ−^(Li)···H^δ+^(N) combination, with the dehydrogenation energy barriers being 145.58 and 239.8 kJ/mol, respectively, indicating that the presence of the –BH_3_ group can reduce the energy barrier of hydrogen release in the system. For the dehydrogenation reactions of LiNH_2_BH_3_–LiH (Path–1–2) and LiNH_2_BH_3_ itself, both follow the dehydrogenation mode of the H^δ−^(B)···H^δ+^(N) combination, with the dehydrogenation energy barriers being 236.95 and 262.81 kJ/mol, respectively, suggesting that the presence of the –LiH group can improve the hydrogen release performance of the system. The presence of –BH_3_ and –LiH groups has a significant effect on the hydrogen release performance of the system. The comparison of the dehydrogenation energy barriers of LiAB, Li2AB and Li2A, the related analysis of the frontier orbitals, and the calculation results of the hydrogen dissociation energy are consistent. The order of the hydrogen dissociation energy of different positions of H atoms in LiAB, Li2AB and Li2A is ΔE_H(N)_ > ΔE_H(B)_ > ΔE_H(Li)_. The dehydrogenation performance of Li2AB is superior to that of LiAB and Li2A.

## 4. Calculation Method

This study utilized the B3LYP hybrid functional method within density functional theory [29,30], combined with the 6–31G(d,p) basis set, to conduct a comprehensive geometry optimization of LiNH_2_BH_3_ and (LiH)_n_ (n = 1–5) clusters, thereby determining their stable conformations. The natural charge distribution of the intermediate 1IM was calculated using the NBO method. The B3LYP method integrates the Lee–Yang–Parr functional and a three–parameter hybrid model by Becke for handling electron exchange [31,32], making it a popular choice in cluster research due to its wide applicability [33,34]. Theoretical calculations and in–depth analyses were carried out to explore the reaction mechanism between LiNH_2_BH_3_ and (LiH)_n_ (n = 1–5) clusters, including the optimization of each stable point along the reaction path. The accuracy of these stationary points and their relationships was verified through frequency analysis and IRC calculations. Frequency analysis confirmed the correctness of the reaction stationary points, showing that the transition state has one imaginary frequency while the other stationary points have none. IRC calculations were utilized to confirm the connectivity of the stationary points along the reaction pathway. Furthermore, we also calculated the relative energies of each reaction stationary point with respect to the reactants and plotted the corresponding energy level diagrams. All calculations were performed using the Gaussian 16, with energy gradient and total energy convergence criteria set to 1 × 10^−6^.

## Data Availability

Dataset available on request from the authors.

## Supplementary Materials

The following supporting information can be downloaded at: https://www.mdpi.com/article/10.3390/molecules30040929/s1, Figure S1: The geometrical configurations of the transition states on the reaction path of LiNH_2_BH_3_ with (LiH)_n_ (n = 1–5); Table S1: Total energies and relative energies at the critical points of potential energy surface and vibrational frequencies.
