# Supplementary material for: Reaction Mechanism Study of LiNH2BH3 and (LiH)n (n = 1–5) Clusters Based on Density Functional Theory
Source: Molecules. 2025 Feb 17;30(4):929. doi: 10.3390/molecules30040929 (PMC11857906; doi:10.3390/molecules30040929)
Supplement: Supplementary file 1 [file molecules-30-00929-s001.zip › molecules-3431486-supplementary.pdf]

Reaction Mechanism Study of $LiNH_2BH_3$ and $(LiH)_n$ (n=1-5) Clusters Based on Density Functional Theory

Xiao Dong, Rong Yuan, Genzhuang Li and Aochen Du*

Xinjiang Laboratory of Phase Transitions and Microstructures in Condensed Matters, College of Physical Science and Technology, Yili Normal University, Yining 835000, China,

1TS1 1TS2 2TS1

2TS2 3TS1 3TS2

4TS1 4TS2

5TS1 5TS2

Figure S1 The geometrical configurations of the transition states on the reaction path of $LiNH_2BH_3$ with $(LiH)_n$ (n=1-5).

Table S1 Total energies and relative energies at the critical points of potential energy surface and vibrational frequencies

Species	E_{total} (a.u.)	E_{rel} (a.u.)	E_{rel} (kJ/mol)	Frequency (cm^{-1})	
1RC	-98.30625	0	0	—	—
1IM	-98.35267	-0.04642	-121.87571	97.8	153.4
1TS1	-98.29722	0.00903	23.708265	-1108.0	161.3
1PC1	-98.31217	-0.00592	-15.54296	52.8	58.8
1TS2	-98.26242	0.04383	115.075665	-1346.7	158.8
1PC2	-98.32763	-0.02138	-56.13319	47.2	58.6
2RC	-106.46322	0	0	—	—
2IM	-106.50264	-0.03942	-103.49721	57.3	87.3
2TS1	-106.45059	0.01263	33.160065	-992.3	69.0
2PC1	-106.47136	-0.00814	-21.37157	45.9	61.8
2TS2	-106.41729	0.04593	120.589215	-1300.1	93.1
2PC2	-106.47886	-0.01564	-41.06282	13.6	15.3
3RC	-114.62191	0	0	—	—
3IM	-114.65454	-0.03263	-85.670065	53.7	125.4
3TS1	-114.59909	0.02282	59.91391	-1111.4	71.0
3PC1	-114.61867	0.00324	8.50662	36.6	66.0
3TS2	-114.57276	0.04915	129.043325	-1350.3	122.5
3PC2	-114.63797	-0.01606	-42.16553	46.4	75.0
4RC	-122.77955	0	0	—	—
4IM1	-122.79493	-0.01538	-40.38019	82.9	114.7
4TS1	-122.75176	0.02779	72.962645	-1110.0	56.8
4PC1	-122.78342	-0.00387	-10.160685	43.2	68.6
4IM2	-122.80512	-0.02557	-67.134035	54.9	106.2
4TS2	-122.72156	0.05799	152.252745	-1322.7	60.6
4PC2	-122.78342	-0.00387	-10.160685	27.2	32.5
5RC	-130.94392	0	0	—	—
5IM1	-130.96419	-0.02027	-53.218885	42.5	101.0
5TS1	-130.91706	0.02686	70.52093	-1122.8	92.5
5PC1	-130.95286	-0.00894	-23.47197	51.2	66.9
5IM2	-130.95378	-0.00986	-25.88743	14.3	67.3
5TS2	-130.86820	0.07572	198.80286	-1350.5	26.8
5PC2	-130.93293	0.01099	28.854245	25.5	41.6
